# Cleaning and disinfection programs against *Campylobacter jejuni* for broiler chickens: productive performance, microbiological assessment and characterization^1^

**DOI:** 10.3382/ps/pex153

**Published:** 2017-07-11

**Authors:** Maria Fernanda de Castro Burbarelli, Gustavo do Valle Polycarpo, Karoline Deliberali Lelis, Carlos Alexandre Granghelli, Agatha Cristina Carão de Pinho, Sabrina Ribeiro Almeida Queiroz, Andrezza Maria Fernandes, Ricardo Luiz Moro de Souza, Maria Estela Gaglianone Moro, Roberto de Andrade Bordin, Ricardo de Albuquerque

**Affiliations:** * Department of Animal Nutrition and Production (VNP), Faculty of Veterinary Medicine and Animal Science, University of São Paulo (FMVZ-USP), Pirassununga, Brazil; † Department of Veterinary Medicine, Faculty of Veterinary Medicine and Animal Science, University of São Paulo (FZEA-USP), Pirassununga, Brazil; ‡ Nutrition, Animal Production, Health - FATEC-SP; § São Paulo State University (Unesp), School of Technology and Agricultural Sciences, Campus of Dracena

**Keywords:** biosecurity, campylobacteriosis, disinfectant, health, poultry

## Abstract

Detailed cleaning and disinfection programs aims to reduce infection pressure from microorganisms from one flock to the next. However, studies evaluating the benefits to poultry performance, the sanitary status of the facilities, and the sanitary quality of the meat are rarely found. Thus, this study was designed to evaluate 2 cleaning and disinfecting programs regarding their influence on productive performance, elimination of *Campylobacter*, and characterization of *Campylobacter jejuni* strains when applied to broiler chickens’ facilities. Two subsequent flocks with 960 birds each were distributed into 32 pens containing 30 birds each. In the first, the whole flock was inoculated with a known strain of *Campylobacter jejuni* in order to contaminate the environment. In the second flock, performance and microbiological evaluations were done, characterizing an observational study between 2 cleaning and disinfection programs, regular and proposed. The regular program consisted of sweeping facilities, washing equipment and environment with water and neutral detergent. The proposed cleaning program consisted of dry and wet cleaning, application of 2 detergents (one acid and one basic) and 2 disinfectants (250 g/L glutaraldehyde and 185 g/L formaldehyde at 0.5% and 210 g/L para-chloro-meta-cresol at 4%). Total microorganism count in the environment and *Campylobacter* spp. identification were done for the microbiological assessment of the environment and carcasses. The positive samples were submitted to molecular identification of *Campylobacter* spp. and posterior genetic sequencing of the species identified as *Campylobacter jejuni*. The birds housed in the facilities and submitted to the proposed treatment had better performance when compared to the ones in the regular treatment, most likely because there was a smaller total microorganism count on the floor, walls, feeders and drinkers. The proposed program also resulted in a reduction of *Campylobacter* spp. on floors, drinkers and birds. Moreover, it was possible to identify 6 different *Campylobacter jejuni* strains in the facilities. The proposed treatment resulted in a positive influence on the birds’ performance and reduction of environment contamination for broiler chickens.

## INTRODUCTION

Preventive practices that include cleaning and disinfection are fundamental steps for biosecurity programs and are indispensable for the maintenance of high productivity of poultry flocks.

The aim of cleaning is the maximum removal of organic matter from facilities and equipment. Therefore, detergents that reduce superficial tension, act in the emulsification of lipids, have dissolving powers on mineral residues and peptizing action on protein residues are utilized. Detergents can be alkaline, acid, or neutral (UGA, 2005). Alkaline detergents have high dissolving power on organic residues; acid detergents have high dissolving power on mineral residues and some organic ones; and the neutral detergents are indicated for delicate surfaces and with weakly adhered residues. The maximum efficacy of disinfection procedures is only possible on surfaces with appropriate removal of organic matter (Ward et al., 2006).

Another fundamental step is disinfection, which aims to reduce infection pressure from microorganism destruction as well as the transmission of pathogens from one flock to the next (UGA, 2005; Tokach et al., 2012). There are a great variety of active ingredients utilized as disinfectants in poultry production such as formaldehyde and glutaraldehyde, which are bactericides, sporicides, and fungicides. Their activity is due to the alkylation of sulfidryl, hydroxyl, carboxyl, and amino groups of microorganisms, altering their DNA, RNA, and protein synthesis. There is also peracetic acid acting as a bactericide by attacking the lipid membrane, DNA, and other cell components through toxic free radicals that a disinfectant produces (Dvorak, 2008). However, cresols have bactericide and viricidal action on the protoplasm of bacterial cells, causing denaturation and protein precipitation (Spinosa et al., 2006).

Production environments with high contamination levels have a direct influence on increased mortality, and/or indirect influence on uniformity and decreased broiler performance (Ristow, 2008; Renaudeau, 2009). Thus, cleaning and disinfection can have a positive influences on the increase in birds’ productive performance, mainly in environments where there is a sanitary challenge (Burbarelli et al., 2015). Besides guaranteeing high productivity, these practices are fundamental to ensure the quality of poultry products, making them appropriate for human consumption.

The occurrence of diseases in humans, transmitted by poultry products, can be related to the birds’ contamination during their life in production facilities, and *Campylobacter* is one of the causative agents of disease (Shane et al., 1986; Stern, 1992; Smith et al., 2008). The contamination of chickens, and consequently of carcasses, is a reason to pay attention to the poultry productive chain (Evans and Sayers, 2000). In the field, one of the goals is the decrease of pathogen colonization in the birds’ intestinal tract since the horizontal transmission of the pathogen is more efficient (Newell and Fearnley, 2003).

However, studies simultaneously evaluating efficiency of cleaning and disinfection protocols, benefits on poultry performance, sanitary status of the facilities and sanitary quality of the meat are rarely found. Thus, the objective of this study was to evaluate 2 cleaning and disinfection programs regarding their effectiveness on broilers’ productive performance and on the elimination and characterization of *Campylobacter jejuni* strains in environments that had been previously contaminated with *Campylobacter jejuni*.

## MATERIAL AND METHODS

### Birds, Installations, and Experimental Scheme

The experimental protocol was approved by the ethics committee for animal experimentation of the Faculty of Veterinary Medicine and Animal Science, University of São Paulo, protocol 3025/2013.

A total of 1,920 day-old male Cobb 500 broiler chicks were divided into 2 subsequent flocks with 960 birds each. In both flocks, the birds were distributed into 32 pens containing 30 birds each. This work was an observational study study between 2 cleaning and disinfection programs: a Regular treatment and a Proposed one.

 The floor was covered with new rice hulls litter and provided with a tubular feeder and bell drinkers. The housing density was 10 birds per m^2^, with average initial weight of 45.5 g ± 0.763 g. The interval between the 2 flocks was 8 d.

The poultry house had average area of 500 m^2^ (considering structure, ceiling, curtains, internal and external parts, paving, flooring, and walls) which was utilized for the analysis and calculation of the cleaning and disinfection program. The poultry house had an internal room that divided it into 2 halves, guaranteeing the isolation of each experimental group.

The diets were formulated with corn and soybean bran according to Rostagno et al. (2011) and provided ad libitum. There was no addition of growth-promoting antibiotics in pre-mixtures. The chicks received chlorinated drinking water at a concentration of 2.0 ppm.

The first-raised broiler group house experiment was designed to create a sanitary challenge by inoculating the birds with *Campylobacter jejuni*. Performance and microbiological evaluations were done in the second flock.

### Sanitary Challenge

In the first housing, on d 11, the chicks were inoculated with standard *Campylobacter jejuni* (ATCC 33560) strains through an oral probe to deposit 1 mL of inoculum consisting of liquid BHI culture medium and 10^5^ UFC/mL of *Campylobacter jejuni*, considered a high dose by Chaveerach et al. (2004).

### Cleaning and Disinfection Programs

Litter and all the equipment were removed from the poultry house to carry out the treatments. In both programs, approximately 0.4 L of diluted solution per m^2^ was used for detergents as well as for disinfectants. The water utilized in the treatments had chlorination at 2.0 ppm.

The Regular treatment was applied in 16 pens and consisted of sweeping facilities, washing equipment (feeder, drinkers, buckets, boots) with neutral detergent, wetting and washing the environment (floors, walls, ceiling, curtains) with water and neutral detergent, and subsequently drying the environment and facilities. The Proposed treatment was also applied in 16 pens and started with cleaning and disinfecting the water supply system: washing the water reservoir and next applying 100 g/kg peracetic acid and 80 g/kg Benzyl-(C12-C16) chloro-alkyl dimethyl ammonium at 0.5%. The product was added to the water reservoir that was posteriorly drained by the water supply system, providing contact with the whole supply system, and kept like that for 12 h, and then drained through the triggers of the bell drinkers. Dry cleaning was done by removing bedding and sweeping the facilities followed by wet cleaning of the house and posterior with pressurized water. All equipment utilized in the poultry house (feeders, drinkers, buckets, boots, trays and other utensils) were washed under pressurized water. Next, alkaline and acid detergents in solution at 4% were applied on all internal and external surfaces (ceiling, walls, flooring, curtains), objects and equipment, followed by rinsing under pressurized water. Disinfectants were only applied after the environment and the equipment were partially dry, without water accumulation. The first utilized disinfectant consisted of 250 g/L glutaraldehyde and 185 g/L formaldehyde at 0.5% and was applied on all surfaces and equipment. The second one, composed of 210 g/L para-chloro-meta-cresol at 4%, was applied only on the floor and walls up to 0.5 m of height. A knapsack sprayer was utilized for both applications.

After finishing the treatments, new bedding was distributed in the pens, the equipment was relocated, and the second group of day-old chicks was distributed in the rearing pens.

### Productive Performance

The birds were weighed on d 1, 7, 21, 35, and 42 of the experiment for performance analysis. The measured response variables in each pen were: body weight gain (**BWG**), feed intake (**FI**), feed: gain ratio (**F:G**), viability (**VB**), and productive efficiency index (**PEI**).

### Microbiological Evaluation

Surface swabs of the floor, wall, drinkers, and feeders were done for total count and evaluation of *Campylobacter* spp. presence from surfaces of 2 cm × 5 cm totaling a 10 cm^2^ area. Samplings of 200 mL of the birds’ drinking water from the bell drinker tap of 5 pens in each treatment were collected. Besides the mentioned points, swabs of 5 birds’ cloaca per program were swabbed for the evaluation of *Campylobacter* spp. presence. The swabs were stored in tubes containing peptone water at 0.1% to maintain colony viability.

The total microorganism count was done 24 h before the cleaning and disinfection procedures and 48 h after them. Plate count agar (**PCA**) was utilized in previously solidified plates with sowing on the surface as described by Evancho et al. (2001).

The evaluation regarding the presence of *Campylobacter* spp. was done in the first house before and after inoculation. In the second flock, the evaluation was done when the birds were 2, 11, and 42 d old. Moreover, harvesting was done right before slaughter, after feathering and after chiller. In each point, 10 carcasses of each experimental group were used.

Direct isolation of *Campylobacter* spp. was done with inoculation of 0.1 mL of peptone water solution at 0.1% in petri dishes containing mCCDA (CM739, Oxoid, Hampshire, England) culture medium with selective supplement (SR155, Oxoid Hampshire, England) where inoculation of 0.1 mL of solution obtained from samples was done. Then they were incubated in an environment whose microaerophilic atmosphere was modified with 5% of O_2_, 10% CO_2_ and 85% N_2_, at 42°C for 48 h in jars for special atmospheres (Probac do Brasil, São Paulo, Brazil). From the positive samples, 1 to 3 possible *Campylobacter* spp. colonies were randomly selected and submitted to Gram staining to differentiate S bacilli from spiral ones. Oxidase and catalase assays were also carried out. The colonies with compatible characteristics to *Campylobacter* spp. morphology were collected for posterior PCR assay.

### Molecular Identification

PCR testing was performed to distinguish *Campylobacter* species in positive samples from the microbiological culture. Five typical colonies from the positive samples of *Campylobacter* spp. were harvested from the same sampling point. The colonies were submitted to bacterial DNA extraction through an adapted thermal shock technique by Fang and Hedin (2003).

Multiplex PCR technique was utilized, based on Klena et al. (2004). Primer pairs of *Campylobacter jejuni, Campylobacter coli, Campylobacter lari*, and *Campylobacter upsaliensis* were utilized for the amplification of DNA fragments found in each of the cited species. The specific primers presented IpxA as the target gene. For *Campylobacter coli*, lpxAColi and lpxARKK2m primers were used; for *Campylobacter jejuni*, lpxAJej and lpxARKK2m; for *Campylobacter lari*, lpxALari and lpxARKK2m; and for *Campylobacter upsaliensis*, lpxAUps and lpxARKK2m.

The amplifications were carried out in 25 μL of solution containing 1.25 μL of reaction buffer 5× Colorless GoTaq Flexi Buffer, 2 μL of MgCl_2_, 0.5 μL of dNTP, 1 μL of each primer (lpxAColi, lpxAJej, lpxALari, lpxAUps), 3 μL of lpxARKK2m primer, 0.25 U of GoTaq DNA Polymerase (Promega, Madison, WI, USA), 11 μL of nuclease-free water, and 3 μL of DNA.

For the amplifications, a thermocycler was utilized, programmed for an initial denaturation cycle at 94°C for 2 min., followed by 40 cycles with denaturation at 94°C, 1 min; annealing at 52°C, 1 min; extension at 72°C, 1 min., and final extension at 72°C, 5 min. The PCR products were analyzed by electrophoresis in agarose gel at 3%, stained with SybrGold (Invitrogen, Karlsruhe, Germany) (0.1 μL/mL) and visualized in a UV trans-illuminator (BioAgency, São Paulo, Brazil). The product sizes were determined by comparing electrophoretic migration standard of a 100-pb molecular size marker (GE Healthcare, USA).

Nuclease-free water was utilized as negative control whereas a strain of *Campylobacter jejuni* (ATCC 33560), the same utilized for environment contamination, was used as positive control.

The samples with electrophoretic migration standard compatible to the positive control were sent to genomic sequencing.

### Genomic Sequencing, Nucleotide Sequence Alignment and Phylogenetic Analysis

DNA fragments were extracted from agarose gel for samples with band size compatible to *Campylobacter jejuni*, and QIAquick Gel Extraction Kit (Qiagen, USA) was utilized after PCR re-amplification following the manufacturer's recommendation.

The sequencing reactions were done utilizing BigDye^®^ Terminator v3.1 Cycle Sequencing Ready Reaction Kit (Applied Biosystems, Life Technologies, USA) containing AmpliTaq DNA Polymerase, according to the manufacturer's specifications, and reactions for sense and anti-sense primers were carried out using an automated sequencer, 3730 DNA Analyzer (Applied Biosystems, Life Technologies, USA).

The search for consensus sequences generated by the program CAP 3 Contig (Huang and Madan, 1999) and edited by BioEdit 7.0.9 (Hall, 1999) was done by BLAST program version 2.0 (Altschul et al., 1997). The editing and multiple alignment of obtained nucleotide sequences as well as others deposited in GeneBank (Table 1) were done by ClustalW program version 1.4 (Thompson et al., 1997), implemented in BioEdit Sequence Alignment Editor version 7.0.9 (Hall, 1999), utilizing default parameters. Distance matrices, given in percentages of similarity/identity, between the nucleotide sequences were calculated through MatGAT program, version 2.0 (Campanella et al., 2003), using global alignment algorithm.

**Table 1.** Utilized nucleotide sequences of *Campylobacter* spp. for phylogenetic reconstruction with genotype, name, origin and respective access numbers in GenBank.

Species	Isolate	Origin	Genbank Access Number
*Campylobacter jejuni*	NCTC 11168	United Kingdom	AL111168
	RM 3668	California	AY531515
	F 38011	Arizona	AY531520
	KLC2851	N. Zeland	AY531522
	RM 3664	California	AY531519
*Campylobacter coli*	RM 1878		AY531493
	RM 1858		AY531495
	RM 1865		AY531494
	RM 1857		AY531496
	WA 27	N. Zeland	AY531510
	RM 3232		AY531504
	RM 1896	USA	AY531492
*Campylobacter Lari*	RM 3659	United Kingdom	AY531477
	RM 2825	Canada	AY531479
	RM 2824	United Kingdom	AY598984
	RM 2819	Canada	AY531485
	RM 2822		AY531482
	RM 2100	USA	AY531474
	RM 2823	Canada	AY531481
	RM 1890		AY531476
*Campylobacter Upsaliensis*	RM 3195	South Africa	AY531473
	RM 2093		AY598987
	RM 2089		AY531472
	RM 1488	Canada	AY531471
Helicobacter hepaticus	ATCC 51449	USA	DN202995

Phylogenetic reconstructions for sequences of 213 nucleotides, related to lpxA gene were done through maximum likelihood algorithm and Jukes and Cantor (**JC**) substitution model with nodal bootstrap support for 1000 pseudo-replicates, utilizing MEGA 5.0 program (Tamura et al., 2013). For the reconstructions, other sequences of *Campylobacter* spp. deposited in GenBank were used as shown in Table 1.

### Statistical Analysis

Data were analyzed by Statistical Analysis System (SAS Institute, 2012). Normality of studentized residual was verified by Shapiro-Wilk's Test PROC UNIVARIATE (SAS Institute, 2012) and the variances compared by Levene's test. The data that did not meet these requirements were submitted to logarithmic transformation. The original or transformed data were submitted to analysis of variance utilizing PROC MIXED (SAS Institute, 2012). The occurrence frequency of *Campylobacter* was analyzed by Chi-square test through PROC FREQ (SAS Institute, 2012). The utilized level of significance was 5% of probability.

## RESULTS

There was no significant effect of treatments in the initial periods (1 to 7, 1 to 21, and 1 to 35 d). However, during the total housing period (1 to 42 d) there was a significant effect of the Proposed treatment, where the birds exhibited greater BWG, FI, F:G, and PEI (Table 2).

**Table 2.** Performance results obtained in the second poultry house - environment submitted to cleaning and disinfection programs.

	Cleaning and disinfection			
Variable	Common	Proposed	SEM	Probability
1-7 d				
BW gain(g)^1^	120	128	0.002	0.054
FI (g)	131	136	0.001	0.202
F:G	1.096	1.081	0.006	0.344
VB (%)	99.79	99.38	0.191	0.300
1-21 d				
BW gain(g)	800	802	0.006	0.865
FI (g)	1493	1504	0.009	0.546
F:G	1.869	1.851	0.010	0.517
VB (%)	99.12	98.90	0.280	0.702
1-35 d				
BW gain(g)	2126	2141	0.019	0.196
FI (g)	3746	3836	0.027	0.106
F:G	1.810	1.781	0.011	0.270
VB (%)	98.13	97.93	0.468	0.830
1-42 d				
BW gain(g)	2447	2610	0.025	0.002
FI (g)	4760	4903	0.033	0.035
F:G	1.958	1.880	0.018	0.050
VB(%)	96.26	95.68	0.715	0.691
PEI	312.0	346.5	5.656	0.001

^1^Body weight (BW), feed intake (FI), Feed:gain ratio (FG), Viability (VB), Productive Efficiency Index (PEI) PEI = (BW × viability/age% × F:G) × 100.

In the total microorganism count, counts were similar before on the floor, wall, drinkers, feeders, and water (Table 3). After the procedures, there was a difference between the treatments, showing that the smallest counts were found in water, feeders, walls, and floor with the Proposed program. The drinkers from different treatments were not different (Table 3).

**Table 3.** Total microorganism count before and after cleaning and disinfection procedures.

	Cleaning and Disinfection			
Sampling point	Common	Proposed	SEM	p*
Before cleaning and disinfection				
Water	5.06	4.34	0.290	0.234
Drinker	6.28	5.56	0.228	0.118
Feeder	4.36	4.68	0.192	0.451
Wall	4.64	5.20	0.297	0.381
Floor	4.94	4.85	0.246	0.881
After cleaning and disinfection				
Water	5.75	2.31	0.664	0.014
Drinker	1.56	0.70	0.380	0.282
Feeder	3.42	0.67	0.563	0.004
Wall	3.10	0.97	0.453	0.008
Floor	3.86	0.14	0.610	<.0001

Data expressed in ufc log10/10 cm^2^ for all variables except for water (ufc log10/ml) *Significance *p* < 0.05.

Table 4 shows the occurrence frequencies of *Campylobacter* spp. There was no difference in the sampled points before and after inoculation. After cleaning and disinfection, there was a smaller occurrence of *Campylobacter* spp. in drinkers and floors with the Proposed program. The birds housed in the facilities after the Proposed treatment also had less *Campylobacter* spp. verified by cloaca swabs 7 d after housing. There were no differences in the occurrence of *Campylobacter* spp. at 42 d old and at slaughter time.

**Table 4.** *Campylobacter* spp. frequency in samples collected throughout the experimental period.

Sampling point	Common	Proposed	p*
Before inoculation			
Drinker	30% (3/10)	50% (5/10)	0.113
Water	30% (3/10)	30% (3/10)	1.000
Bird	40% (4/10)	30% (3/10)	0.490
Floor	20% (2/10)	10% (1/10)	0.490
After inoculation			
Drinker	30% (3/10)	50% (5/10)	0.113
Water	30% (3/10)	40% (4/10)	0.113
Bird	20% 92/10)	40% (4/10)	0.196
Floor	10% (1/10)	0% (0/10)	0.291
Wall	10% (1/10)	0% (0/10)	0.291
Feeder	10% (1/10)	0% (0/10)	0.291
After cleaning and disinfection			
Drinker	30% (3/10)	0% (0/10)	0.038
Water	10% (1/10)	10% (1/10)	1.000
Bird	30% (3/10)	0% (0/10)	0.038
Floor	30% (3/10)	0% (0/10)	0.038
42 d			
Drinker	40% (4/10)	30% (3/10)	0.490
Water	40% (4/10)	20% (2/10)	0.525
Bird	30% (3/10)	50% (5/10)	0.291
Floor	10% (1/10)	10% (1/10)	1.000
Wall	10% (1/10)	10% (1/10)	1.000
Slaughter			
Live birds	7,5% (3/40)	20% (8/40)	0.076
Feathering	20% (4/20)	5% (1/20)	0.121
Chiller	10% (2/20)	10% (2/20)	1.000

* *Chi-square test with significance level of 5% (*P* < 0.05).

After the contamination frequency analysis, the positive samples were submitted to PCR and genomic sequencing, totaling 54 samples. The sequencing results confirmed *Campylobacter jejuni* for approximately 43% (23/54) of the samples. Besides the samples identified as *Campylobacter jejuni*, other enterobacteria were found after sequencing, such as *Enterobacter cloacae, Enterobacter asburiae*, and *E. coli*.

Table 5 presents the percentages of similarity (under the diagonal) and identity (over the diagonal) of nucleotide sequences found among the selected and sequenced samples and the reference sequences for *Campylobacter* deposited in GenBank (Table 3). The high similarity level among the samples obtained in this study can be observed, varying from 99.1 to 100%. Moreover, all the samples have a high similarity level of 98.6% with the standard ATCC 33560 strain. The same occurs with the identity among the samples, 99.1 to 100% among samples and 98.6% with standard strain.

**Table 5.** Comparison of similarity (under diagonal) and identity (over diagonal) percentages of nucleotide sequences of 331pb fragments of *Campylobacter jejuni* IpxA gene, among 6 samples detected, posteriorly sequenced, by multiplex PCR and sequences of other *Campylobacter* recovered in GenBank.

	Similarity/Identity (%)															
	BR597	BR212	BR1241	BR1236	BR1206	BR1037	RM3668	RM3664	NCTC11168	KLC2851	F38011	ATCC33560	RM2825	RM1878	RM3195	ATCC51449
Isolated	Cjej	Cjej	Cjej	Cjej	Cjej	Cjej	Cjej	Cjej	Cjej	Cjej	Cjej	Cjej	Clari	Ccoli	Cups	Hhepaticus
BR597Cjej	–	100.0	99.1	99.1	99.1	99.1	99.1	98.6	99.1	99.1	99.5	98.6	76.1	86.4	77.0	52.8
BR212Cjej	100.0	–	99.1	99.1	99.1	99.1	99.1	98.6	99.1	99.1	99.5	98.6	76.1	86.4	77.0	52.8
BR1241Cjej	99.1	99.1	–	100.0	100.0	100.0	100.0	98.6	99.1	99.1	99.5	98.6	76.5	86.4	77.0	52.8
BR1236Cjej	99.1	99.1	100.0	–	100.0	100.0	100.0	98.6	99.1	99.1	99.5	98.6	76.5	86.4	77.0	52.8
BR1206Cjej	99.1	99.1	100.0	100.0	–	100.0	100.0	98.6	99.1	99.1	99.5	98.6	76.5	86.4	77.0	52.8
BR1037Cjej	99.1	99.1	100.0	100.0	100.0	–	100.0	98.6	99.1	99.1	99.5	98.6	76.5	86.4	77.0	52.8
RM3668Cjej	99.1	99.1	100.0	100.0	100.0	100.0	–	98.6	99.1	99.1	99.5	98.6	76.5	86.4	77.0	52.8
RM3664Cjej	98.6	98.6	98.6	98.6	98.6	98.6	98.6	–	99.5	99.5	99.1	100.0	76.5	86.9	77.0	54.2
NCTC11168Cjej	99.1	99.1	99.1	99.1	99.1	99.1	99.1	99.5	–	100.0	99.5	99.5	77.0	86.4	77.5	53.7
KLC2851Cjej	99.1	99.1	99.1	99.1	99.1	99.1	99.1	99.5	100.0	–	99.5	99.5	77.0	86.4	77.5	53.7
F38011Cjej	99.5	99.5	99.5	99.5	99.5	99.5	99.5	99.1	99.5	99.5	–	99.1	76.5	86.9	77.5	53.2
ATCC33560Cjej	98.6	98.6	98.6	98.6	98.6	98.6	98.6	100.0	99.5	99.5	99.1	–	76.5	86.9	77.0	54.2
RM2825Clari	76.1	76.1	76.5	76.5	76.5	76.5	76.5	76.5	77.0	77.0	76.5	76.5	–	71.4	71.2	53.7
RM1878Ccoli	86.4	86.4	86.4	86.4	86.4	86.4	86.4	86.9	86.4	86.4	86.9	86.9	71.4	–	76.5	50.7
RM3195Cups	77.0	77.0	77.0	77.0	77.0	77.0	77.0	77.0	77.5	77.5	77.5	77.0	71.8	76.5	–	54.6
ATCC51449Hhep	53.5	53.5	53.5	53.5	53.5	53.5	53.5	54.9	54.5	54.5	54.0	54.9	54.5	51.2	55.4	–

When the samples (Table 6) were compared to other species of *Campylobacter*, there was smaller similarity and identity, which ranged from 69 to 86.9% for both. When compared to *Helicobacter hepaticus*, the values were 53.5% for similarity and 52.8% for identity.

**Table 6.** Legends of sequenced samples utilized to build the phylogenetic tree.

Identification	Sampling point	Gene Bank Accession n°
BR212Cjej	Bird 12 –initial environment	KY321332
BR597 Cjej	Bird 3 –After inoculation	KY321333
BR1241Cjej	Bird - 42 d Proposed Program	KY321334
BR1236Cjej	Bird 30–42 d Common Program	KY321335
BR1037Cjej	Bird 1 - Feathering Proposed Program	KY321336
BR1206Cjej	Bird 2 Chiller - Proposed Program	KY321337

**Figure 1.**
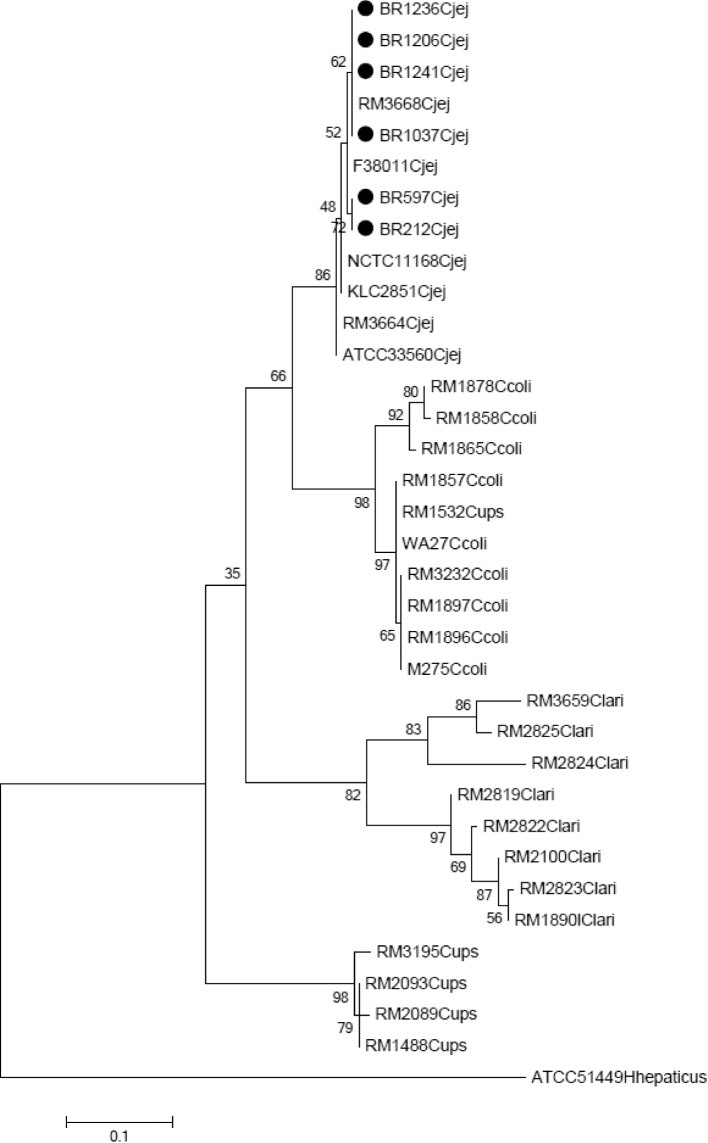
Cladogram representing phylogenetic reconstruction based on nucleotide sequence alignment referring to the amplification of a 340 pb fragment of *Campylobacter* IpxA gene.

Figure 1 illustrates the cladogram obtained in phylogenetic nucleotide reconstruction for *Campylobacter* sequences. The sequenced samples in this study were grouped into 2 subclades of *Campylobacter jejuni*. The samples and the standard ATCC 33560 strain are part of the same monophyletic clade. The bootstrap number of samples varied from 62 to 72%.

## DISCUSSION

High bacterial populations are responsible for a decrease in broiler chickens’ performance (Payne et al., 2005). Thus, cleaning and disinfection practices have positive effects on broilers’ performance (Sharma, 2010) and on prevention of disease (Cozad and Jones, 2003; Newel et al., 2011); however, there are few studies that directly relate cleaning and disinfection to poultry's performance characteristics. The positive effects presented in this study corroborate the ones by Burbarelli et al. (2015), who observed an improvement in broilers’ productive performance and reduction in microorganism count with a detailed cleaning and disinfection program for a flock with reutilized bedding. Bragg and Plumstead (2003) and Ka-Oud et al. (2008) also found beneficial effects of cleaning and disinfection such as greater final weight and lower mortality rate.

In poultry, the satisfactory performance expression is related to intestinal health (Mayorka et al., 2002); therefore, the microbiota balance is an important factor for productivity. Approximately 20% of ingested crude energy is spent on the maintenance of the intestinal epithelium. In addition, the reduction in nutrient absorption has negative influences on F:G, carcass yield, and production cost (Hoerr, 2001). Cleaning and disinfection practices are responsible for reduction of environment infection pressure (Sesti et al., 1998), favoring the intestinal microbiota balance, increasing nutrient absorption and consequently resulting in better expression of broiler chickens’ genetic potential.

The decrease in total microorganism count in equipment and facilities as a result of the Proposed treatment is in accordance to the studies by Luyckx et al. (2015), who observed a greater decrease in total microorganism count when a wet phase was included in the environment. This seems to be related to the easy organic matter removal of microorganisms with high-pressure washing (Grezzi, 2008). Organic matter removal seems to be fundamental to cleaning and disinfection programs because their residues are able to decrease the action of disinfectants (Stringfellow et al., 2009; Chima et al., 2012; Luyckx et al., 2017), resulting in greater microorganism counts even after disinfection.

Fewer positive samples of *Campylobacter* spp. were observed from drinkers, floor, and birds after the Proposed program, corroborating Van de Gissen et al. (1998) and Newell and Fearnley (2003) regarding the reduction capacity and elimination of *Campylobacter* spp. from the environment; however, that contradicts Bouwknegt et al. (2004), who did not find any effect of this type of treatment on facilities for broiler chickens.

Although it did not differ between the analyzed groups, the occurrence of *Campylobacter* spp. in the birds’ drinking water even after the Proposed program deserves attention. The parts of the water provision system of broiler chickens’ houses were a location of biofilm formation due to constant water contact (Araújo et al., 2011). Bacteria such as *Campylobacter* frequently are associated with biofilm (Shi and Szu, 2009). Because it is a gram-negative bacterium, *Campylobacter* is more resistant to the action of disinfectants (Dahl et al., 1989), through a complex enzymatic system of resistance to oxidative stress, and among the enzymes of this system are superoxide dismutase, catalase and cytochrome C peroxidase (Atack and Kelly, 2009). The disinfectants utilized in the water system disinfection were peracetic acid and benzalkonium chloride, the former is an oxidant agent and the latter a quaternary ammonium compound.

Besides the bacterial resistance to the active ingredient we used, biofilm represents an additional resistance to bacteria (Chapman, 2003), because it is able to form a protection through its compounds, making oxidant compounds be inactivated even before getting in contact with the microorganisms (Chen and Stewart, 1996). Efficiency reduction of peracetic acid, an oxidant agent, was also observed by Trachoo and Frank (2002) against *Campylobacter* in the presence of biofilms. The same authors also observed that quaternary ammonium compounds such as benzalkonium chloride have their efficiency affected as well. These factors can be related to re-colonization of facilities by *Campylobacter* after the Proposed treatment when the birds are 42 d old.

The absence of *Campylobacter* spp. in the other samples right after the Proposed treatment of cleaning and disinfection may be related to low resistance of *Campylobacter* spp. to glutaraldehyde and formaldehyde as found by Wang et al. (1983) and Gutiérrez-Martín et al. (2011).

At 42 d old, there was no difference between the contamination frequency of *Campylobacter* for both treatments, which can be related to the high dissemination capacity of these bacteria, as Knudsen, et al. (2006) observed in their studies. Contamination by *Campylobacter* was found in samples from the environment, considered negative for *Campylobacter* spp. as also reported by Van de Gissen et al. (1998). When investigating the contamination origin of those samples, the same authors found the bacteria in insects and staff shoes, suggesting that even the facilities that were previously free from *Campylobacter* spp. can be contaminated during the birds’ stay due to external sources.

Overall, the absence of sanitizing procedures can be considered a *Campylobacter* spp. contamination risk factor for broiler chickens (Evans and Sayers, 2000; Newell and Fearnley, 2003; Bouwknegt et al., 2004; McDowell et al., 2008; Newell et al., 2011), but even with efficient strict programs of cleaning, disinfection and biosafety, this bacterium may enter the facilities and colonize the birds (Van de Gissen et al., 1998).

There is a noteworthy relation between the rearing environment contamination and *Campylobacter* spp. presence in broiler chickens’ carcasses because facilities with *Campylobacter*-positive birds generate positive carcasses (Herman et al., 2003). Elvers et al. (2011) found little changes in the profile of the strains found in carcasses from flocks that were positive for *Campylobacter*, indicating that if the contaminated flock has reached the slaughter plant, there will be little or no influence on the sanitary quality improvement of those carcasses.

Although no significant difference was found in the contamination frequency of 42-day-old birds and carcasses in different slaughter points between the evaluated treatments, it is important to point out that cleaning and disinfection must be adopted to reduce the risk of campylobacteriosis risk in consumers of poultry products (Van de Gissen et al., 1998; Gibbens et al., 2001; Vandeplas et al., 2008; Meunier et al., 2015).

In positive samples of *Campylobacter* submitted to PCR analyses and posterior genetic sequencing, it was possible to detect *Campylobacter jejuni*, and *Enterobacter cloacae, Enterobacter asburiae* and *E. coli*, which should have had their growth inhibited by the utilization of selective supplement mCCDA culture medium. Chon et al. (2011) observed low sensitivity and selectivity to this medium, mainly when the sample microflora was abundant. Bolton et al. (1996) found 13% of contamination of this medium when evaluating samples of human feces.

The utilized multiplex PCR was based on the methodology proposed by Klena et al. (2004), which uses lpxA gene in species differentiation of *Campylobacter spp*. (*C. jejuni, C. coli, C. lari*, and *C. upsaliensis*) that codifies Lpxa enzyme, the initial step of lipid A production, an essential molecule of LPS system found in bacteria of *Campylobacter* genus. This gene, found in several gram-negative bacteria (Weckesser and Mayer, 1988), was identified in *Neisseria meningitidis* (Odegaard et al., 1997), *Pseudomonas aeruginosa* (Dotson et al., 1998), *Enterobacter asburiae* (Osei Sekyere et al., 2016), *Enterobacter cloacae* (Mcgann et al., 2015) and *Escherichia fergusonii* (Touchon et al., 2009).

The identification of *Enterobacter cloacae, Enterobacter asburiae*, and *E. coli* can be related to the presence of lpxA gene in these bacteria and there is also the possibility of genetic information exchange between the environmental microbiota bacteria through plasmids, transposons and gene insertion sequences. Fouts et al. (2005) when studying the genome of some *Campylobacter* strains, found plasmids involved in the transfer and secretion of virulence factors, sequences of chromosome and plasmid DNA insertion.

As the utilized gene, lpxA, belongs to LPS virulence factor of *Campylobacter*, there is the probability of lateral genetic transfer occurrence, which makes it possible that the primers utilized in multiplex PCR reaction, specific for *Campylobacter* species (*jejuni, coli, lari*, and *upsaliensis*), have interacted with similar sequences, but with other bacteria.

Moffat et al. (2011) when studying *Acinetobacter baumannii*, a bacterium that possess lpxA gene, found a gene insertion element of this same gene, showing that bacteria that have it can perform lateral genetic transfers. In this same study, the studied insertion sequence was related to resistance to antibiotics against *Acinetobacter baumannii*, which means a serious public health problem.

In *Campylobacter*, LPS composition, codified by several genes, including lpxA, is also related to resistance to antibiotics (Van Mourik et al., 2010). Thus, it is important to point out that as reported for *A. baumannii* (Potron et al., 2015), *Campylobacter* can have an important role in the dissemination of genes resistant to antibiotic against other gram-negative microorganisms when they may transfer genes laterally.

Evaluating the phylogenetic tree and the similarity and identity table, it is possible to verify that the sample strains are very similar among themselves, are very close to one another in cladogram for lpxA gene, and have high similarity and identity indices. When comparing the samples to the standard strains, there was a slight distancing in the cladogram and lower similarity and specificity indices, showing that it is small despite the differences among these strains.

The high level of similarity and specificity found in this study are in accordance with Lucien (2012) when evaluating the tuf gene of these bacteria, with similarity and identity varying from 98 and 99% of *Campylobacter jejuni* samples with standard ATCC 33560 strain.

In the present study, it was possible to observe 4 distinct clades for the phylogenetic reconstruction of *Campylobacter* spp, one for each studied species of *Campylobacter, Campylobacter jejuni, Campylobacter coli, Campylobacter lari*, and *Campylobacter upsaliensis*, similarly to Kärenlampi et al. (2004), Klena et al. (2004) Hill et al. (2006) and Lucien (2012) when studying groEL gene, lpxA gene, tuf gene, and cpn60 gene, respectively.

Our results showed that the strains of *Campylobacter jejuni* circulating the assessed experimental environment are very similar to the ones that could be found in different countries and types of samples, making it possible to observe that even with a great diversity of *Campylobacter jejuni* strains, they have genetic similarity among themselves, making the utilized methodology reliable for their identification and phylogenetic reconstruction.

Although the recovery of standard ATCC 33560 strains in the samples was expected, no sample could be identified after sequencing. This result can be related to a reduced adaptation to the environment and competition with the other *Campylobacter jejuni*, inhibiting a possible growth of standard ATCC 33560 strain. Cawthraw et al. (1996) reported the fragility of bacteria from in vitro cultures, since the laboratory conditions in which they are submitted differ from the in vivo environment, reducing their resistance, adhesion factors, motility, and virulence (Ringoir and Korolik, 2003). There is also the possibility of genomic rearrangement occurrence, insertions, deletions, or mutations of *Campylobacter jejuni* DNA as described by Wassenaar et al. (1998), Hänninen et al. (1999), and De Boer et al. (2002), resulting in modifications of these same factors.

## CONCLUSION

The Proposed program shows greater efficiency in the total environmental microorganism count reduction and the capacity to eliminate *Campylobacter* from the floor and drinkers of facilities. With the adoption of the Proposed treatment, it is possible to obtain better performance of the birds. Both programs do not influence the occurrence frequency of *Campylobacter* in the facilities and birds at 42 d old and at slaughter time. It was possible to identify 6 different strains of *Campylobacter jejuni*, which occupy the same phylogenetic clade, and become effectively differentiated with high values of nodal support associated with other species of *Campylobacter* to which they were compared.
